# The Association of Flavin Mononucleotide and Lactate During Normothermic Regional Perfusion with Post-Transplant Liver Outcomes: An Exploratory Study

**DOI:** 10.3390/medicina62091622

**Published:** 2026-08-22

**Authors:** Raphaël M. J. Fischer, Claire Cywes, Olivia Ong, Nicolas Muñoz, Sardar Din, Tobenna Ibeabuchi, Isabela Brown-Soler, Nadim Mahmud, Kelly Cederquist, Georgeine Smith, Stephanie Benko, Chase J. Wehrle, Elizabeth M. Sonnenberg, Matthew H. Levine, Samir Abu-Gazala, James F. Markmann, Kim M. Olthoff, Abraham Shaked, Frederick Vyas, Marty Sellers, Jeroen de Jonge, Keyue Sun, Andrea Schlegel, Peter L. Abt

**Affiliations:** 1 Department of Surgery, Penn Transplant Institute, University of Pennsylvania, Philadelphia, PA 19104, USA; olivia.ong@pennmedicine.upenn.edu (O.O.); nicolas.munoz@pennmedicine.upenn.edu (N.M.); sardarnaseer@gmail.com (S.D.); isabela.brown-soler@pennmedicine.upenn.edu (I.B.-S.); nadim.mahmud@pennmedicine.upenn.edu (N.M.); kelly.cederquist@pennmedicine.upenn.edu (K.C.); georgeine.smith@pennmedicine.upenn.edu (G.S.); stephanie.benko@pennmedicine.upenn.edu (S.B.); elizabeth.sonnenberg@pennmedicine.upenn.edu (E.M.S.); matthew.levine@pennmedicine.upenn.edu (M.H.L.); samir.abu-gazala@pennmedicine.upenn.edu (S.A.-G.); james.markmann@pennmedicine.upenn.edu (J.F.M.); kim.olthoff@pennmedicine.upenn.edu (K.M.O.); abraham.shaked@pennmedicine.upenn.edu (A.S.); frederick.vyas@pennmedicine.upenn.edu (F.V.); peter.abt@pennmedicine.upenn.edu (P.L.A.); 2 Erasmus MC Transplant Institute, Erasmus Medical Center, Dr. Molewaterplein 40, 3015 GD Rotterdam, The Netherlands; j.dejonge.1@erasmusmc.nl; 3 Mayo Clinic Alix School of Medicine, Mayo Clinic, Rochester, MN 55905, USA; 4 School of Medicine, Meharry Medical College, Nashville, TN 37208, USA; tibea927@mmc.edu; 5 Transplant Center, Digestive Disease & Surgery Institute, Cleveland Clinic, Cleveland, OH 44195, USA; wehrlec@ccf.org (C.J.W.); sunk2@ccf.org (K.S.); schlega4@ccf.org (A.S.); 6 Gift of Life Donor Program, Philadelphia, PA 19123, USA; msellersmd@gmail.com

**Keywords:** flavin mononucleotide, FMN, normothermic regional perfusion, NRP, organ donation, organ preservation, DCD

## Abstract

*Background and Objectives*: Normothermic regional perfusion (NRP) is an effective technique to improve recipient outcomes. However, no valid methods for determining liver viability exist. Identifying relevant biological markers with clinical correlation to liver viability and recipient clinical outcomes is imperative to improve decision-making. *Materials and Methods*: Fifty-four donors undergoing NRP and their recipients were included. FMN was measured at the start, 1 h, and end of NRP. Lactate was measured at the start, 30, 60, and 90 min of NRP. Primary outcomes were early allograft function using the early allograft dysfunction (EAD) criteria and the model for early allograft function (MEAF). Secondary outcomes included the association with donor warm ischemia time (DWIT), stratified by 30 min. Spearman’s rho assessed correlations. *Results*: Forty (74%) livers were transplanted, with 5 (12.5%) recipients developing EAD and the mean MEAF score was 3.13 (1.49). FMN at all time points was significantly greater in the EAD group compared to the non-EAD group and was significantly correlated with the MEAF score (Peak FMN: rho = 0.507, *p* = 0.001). FMN during perfusion did not differ between DWIT < and ≥30 min. Lactate levels were similar across all groups and did not discriminate for adverse allograft outcomes. However, lactate levels during perfusion were higher in the DWIT ≥ 30 min group. *Conclusions*: These findings suggest that during NRP, elevated FMN, but not lactate, is associated with early allograft function.

## 1. Introduction

Normothermic regional perfusion (NRP) is an effective technique to expand donation after circulatory death (DCD) liver utilization and improve recipient outcomes [1,2,3,4,5]. However, there are no valid methods for determining liver viability or assessing ischemic damage associated with the obligatory period of donor warm time. A variety of approaches to evaluate the viability of DCD NRP donor livers have been described, including assessing graft appearance during perfusion, bile production and biochemistry, hepatocellular enzymes in the perfusate, and circuit flow characteristics [6,7,8]. However, to a large degree, decision-making relies upon trends of lactate clearance or at least lactate stability, although the data to support this approach is limited [9]. During NRP, multiple organs and tissues are perfused. Lactate levels are therefore not only influenced by hepatic metabolism, but also the release of lactate from ischemic tissues as well as exogenous lactate derived from blood transfusions that may be added to the circuit during NRP. As such, identifying relevant biological markers with robust clinical correlation to liver viability and recipient clinical outcomes is imperative to improve decision-making.

In recent years, flavin mononucleotide (FMN) has been proposed as a promising biomarker for organ injury [10,11,12,13]. FMN is an auto-fluorescent coenzyme noncovalently bound to mitochondrial complex I in the electron transport chain. FMN serves as the primary electron acceptor from NADH, which is transferred to ubiquinone, ultimately resulting in proton pumping from the mitochondrial matrix to the intermembrane space. Several decades ago, it was recognized that when oxygen is reintroduced in ischemic tissue, FMN dissociates and is released from its pocket in complex I [14,15,16,17]. FMN has been quantified in various tissues and fluids after ischemia–reperfusion injury, including machine perfusates. Measurement of FMN during ex situ machine perfusion of the liver in either hypothermic or normothermic conditions has demonstrated potential as a method to assess graft viability and utility with respect to making a decision whether or not to transplant the liver [10,18,19]. However, there exist little data with respect to measuring FMN during NRP and whether systemic FMN has utility in assessing liver allograft viability. We hypothesize that if FMN levels were able to guide utilization of DCD livers during NRP, livers with inferior outcomes could be avoided. In this initial exploratory study, we sought to measure perfusate levels of FMN and lactate during NRP, compare the changes during perfusion and correlate FMN and lactate levels with liver allograft outcomes.

## 2. Materials and Methods

### 2.1. Study Design and Cohort

This was a single-center retrospective analysis conducted from February 2022 to July 2025 of Maastricht type-III donors undergoing NRP and the respective liver recipients. Donors underwent thoracoabdominal (TA) or abdominal (A) NRP at the discretion of the organ procurement organization, donor hospital, and/or donor family. TA-NRP involved clamping and venting of the arch vessels, sequential cannulation of the right atrium and ascending aorta, and initiation of NRP. A-NRP involved clamping and venting of the descending thoracic aorta, sequential cannulation of the abdominal IVC or iliac vein and abdominal aorta or iliac artery, and initiation of NRP [20]. Research authorization was obtained from all donors and/or next-of-kin. The study was approved by the Hospital of the University of Pennsylvania institutional review board (IRB) on 4 June 2021 with approval reviews performed on 24 April 2024 and 13 March 2026, as well as from the Gift of Life Medical Board.

### 2.2. Exposure

Donor demographic data, pre-withdrawal laboratory values, arterial lactate, and donor hemodynamics were collected. Lactate levels were assessed at the start of perfusion (T0), 30 min (T30), 1 h (T60), and 90 min (T90). Donor warm ischemia time (DWIT) was defined as the time from extubation to the establishment of NRP and functional donor warm ischemia time (fDWIT) as the time from mean arterial pressure less than 60 mmHg to the establishment of NRP. DWIT and fDWIT were stratified by length of ischemia, with a cut-off of 30 and 20 min, respectively [21,22]. Recipient WIT was defined as the start of liver implantation until portal reperfusion. Total WIT was defined as DWIT plus recipient WIT. The Model for End-Stage Liver Disease with sodium (MELD-Na) was calculated for recipients. Post-transplant laboratory values during the first postoperative week included aspartate aminotransferase (AST), alanine aminotransferase (ALT), creatinine and bilirubin. Early allograft dysfunction (EAD) was defined as proposed by Olthoff et al. [23]. The model for early allograft function (MEAF) was calculated using the previously described formula [24]. Biliary complications included anastomotic strictures (AS), ischemic cholangiopathy, and biliary leakage (BL). Acute kidney injury (AKI) was defined as a two-fold increase in creatinine during the first postoperative week when compared to the preoperative serum creatinine value or the initiation of renal replacement therapy in the first week after transplant [25].

### 2.3. Outcomes

The primary outcome was the association of FMN and lactate with early allograft function. Secondary outcomes included the association of FMN and lactate with donation characteristics, laboratory values during the first post-operative week, and the occurrence of AKI and biliary complications.

### 2.4. Liver Viability Assessment

Viability assessment of the liver during perfusion was done by a combination of macroscopic assessment of the liver appearance, assessment of the lactate clearance, and adequate perfusion. While no standardized protocol was used, factors associated with non-utilization of the liver were increasing lactate levels and/or diseased appearance of the liver and/or poor perfusion parameters. Assessment of FMN was not performed as part of the decision-making process.

### 2.5. Perfusate FMN

NRP perfusate was sampled from the arterial port at the start (T0), 1 h (T1), and end of perfusion (T2). Duration of perfusion was targeted at 1.5 to 2 h; however, NRP duration was often determined with input from the thoracic team, organ procurement organization (OPO), and the donor hospital. Absence of T2 FMN determination reflects variations in NRP duration. Perfusate samples were immediately stored on ice. Upon arrival to the recipient center, the blood was centrifuged at 5000× *g* for 5 min, plasma was collected and stored at −80 °C until analysis. The plasma FMN concentration was determined by fluorescence spectroscopy using a BioTek Synergy™ H1 Multimode Reader (Santa Clara, CA, USA) according to a previously published protocol [13].

### 2.6. Statistical Analysis

Descriptive statistics were presented for the total donor cohort as well as stratified by transplanted vs. non-utilized status. To assess normality of continuous data, histograms were visually assessed and the Shapiro–Wilk test was performed. Parametric continuous data were presented as mean ± standard deviation and non-parametric continuous data were presented as median interquartile range (IQR). Stratified comparisons were made using *t*-tests and Mann–Whitney U tests as indicated. For parametric data, Student’s *t*-test was used. Comparison of categorical data utilized the Chi-squared or Fisher’s exact test, depending on expected cell counts. Recipient characteristics were summarized similar to the above. Next, median FMN and mean lactate levels were plotted across timepoints and stratified by (1) utilized vs. non-utilized and (2) TA-NRP vs. A-NRP. To compare longitudinal paired data, the Wilcoxon-signed rank test was used. Between-group comparisons were performed using *t*-tests or Mann–Whitney U tests, as indicated. FMN and lactate levels over time were also evaluated across stratified groups with presence or absence of postoperative complications of interest, as well as across ischemic time groupings. Delta FMN and delta lactate were calculated to assess changes between T0 and each subsequent time point. Correlations were examined with Spearman’s rank test for non-parametric data and Pearson correlation for parametric data with binary variables. All analyses were performed with Stata (version 18.1, STATA Corp, College Station, TX, USA).

## 3. Results

### 3.1. Cohort and Perfusion Characteristic

A total of 54 donors with a median age of 46 [38–55] years underwent NRP (Table 1). Of these donors, 30 (56%) were male, 31 (57%) underwent TA-NRP, and 23 (43%) underwent A-NRP. 40 (74%) of the livers were utilized for transplantation. Declination of the grafts was due to high or rising lactate in 8 donors (57%), diseased appearance of 3 grafts (21%), and other reasons including prolonged WIT in 3 donors (21%). Donors with non-utilized livers had significantly greater pre-withdrawal levels of bilirubin (0.70 mg/dL [0.60–1.10] vs. 0.45 mg/dL [0.30–0.60], *p* = 0.013). Non-utilized livers experienced longer fDWIT (24 min [20–31] vs. 19 min [16–24], *p* = 0.009). The median total WIT and cold ischemia time (CIT) were 81 [76–88] and 281 [227–322] minutes, respectively (Table 2). Seven livers subsequently underwent ex situ machine perfusion prior to transplantation due to recipient logistics. The median recipient age was 58 [50–64] years and 23 (57%) were female (Supplementary Table S1). The median MELD score was 23 [16–26]. A total of 5 (12.5%) recipients developed EAD and the mean MEAF score was 3.13 (1.49). 13 (32.5%) recipients developed AKI of which 6 (46%) recipients required dialysis and 4 (10%) recipients experienced biliary complications (3 anastomotic strictures; 1 bile leak). No recipients developed ischemic cholangiopathy. From the livers placed on ex situ machine perfusion, 1 graft developed EAD and no grafts developed biliary complications. The median follow-up time was 456 [209–644] days.

### 3.2. FMN Levels During Perfusion and Association with Outcomes

**Figure 1:**
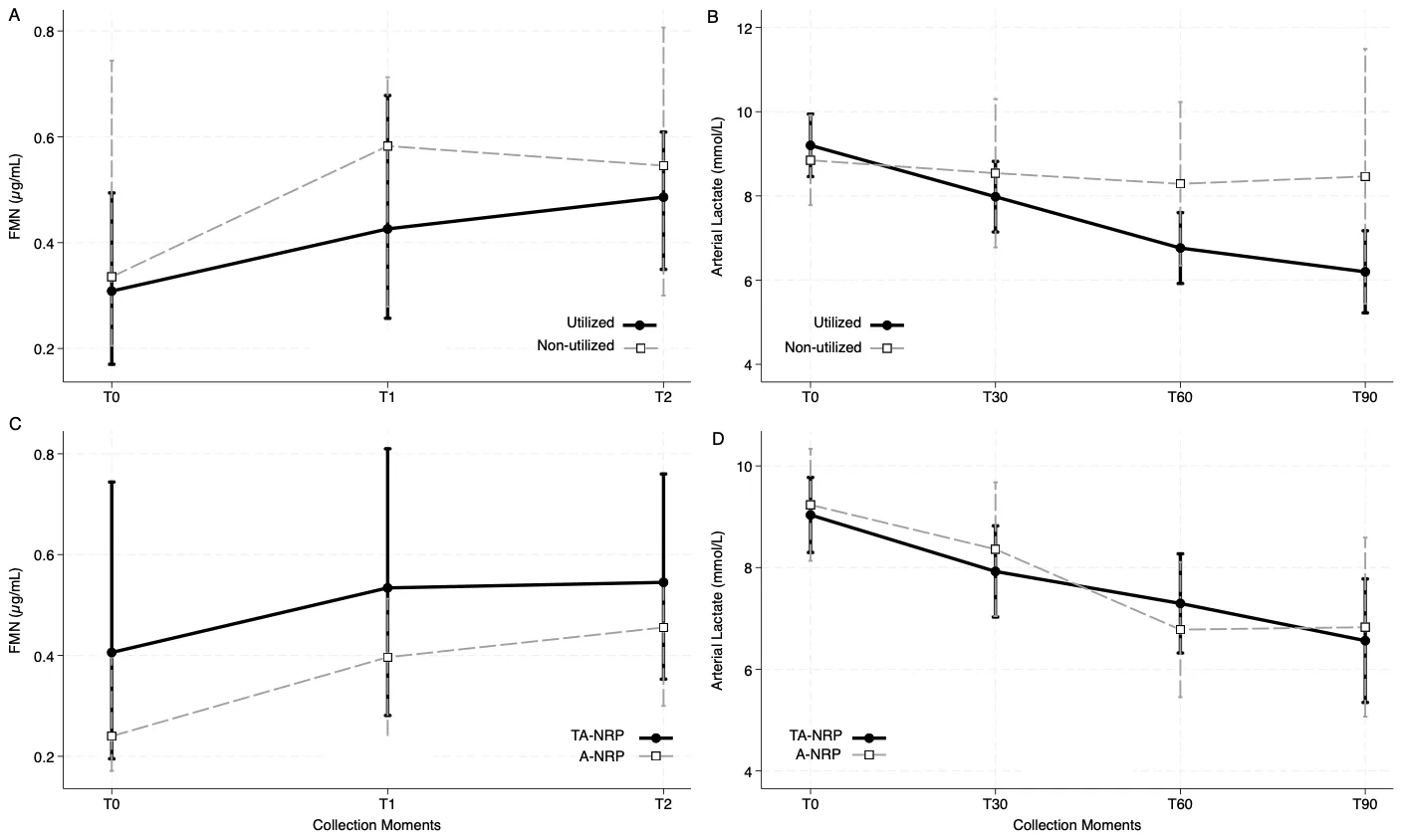
Median FMN (**A**) and mean lactate (**B**) levels between donors with transplanted or non-utilized livers, and between FMN (**C**) and lactate (**D**) in donors undergoing TA-NRP or A-NRP.

FMN at T0 was collected at 5 [3–11] minutes, FMN at T1 at 62 [60–71], and FMN T2 at 110 [96–119] minutes of perfusion. Median FMN levels increased from 0.309 µg/mL [0.183–0.575] at T0 to 0.443 µg/mL [0.260–0.678] at T1 (*p* < 0.001). At T2, the median FMN value increased to 0.486 µg/mL [0.345–0.686] from T0 (*p* < 0.001) and T1 (*p* = 0.025) (Table 3 and Figure 1). Median peak FMN was 0.520 µg/mL [0.344–0.797]. In donors with utilized livers, FMN decreased during the first hour in 9 (22.5%), increased in 30 (75%) and was missing in 1 (2.5%).

**Figure 2:**
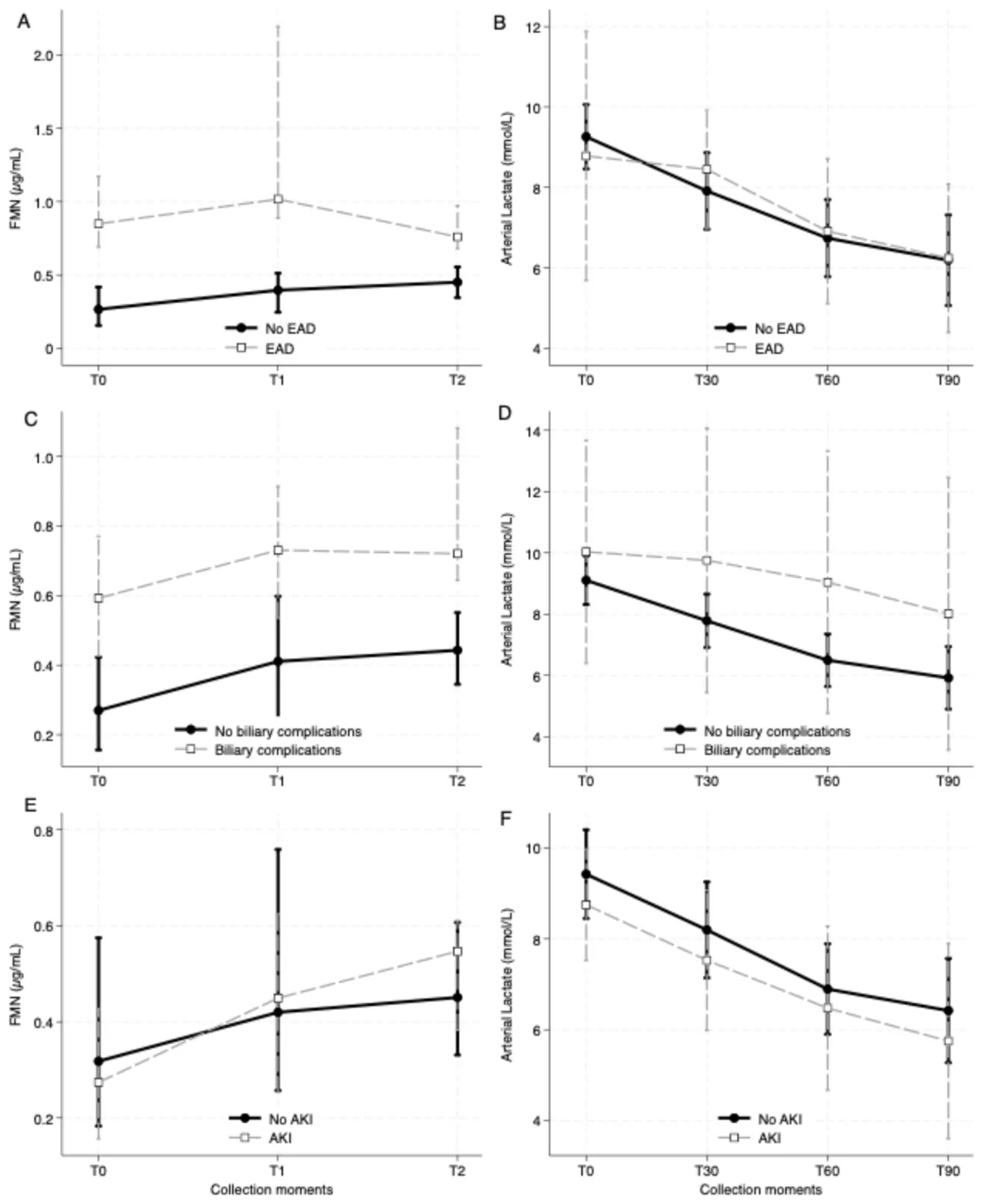
Median FMN (**A**) and mean lactate (**B**) levels from donors where the recipient developed early allograft dysfunction (EAD), (**C**,**D**) donors where the recipients developed biliary complications, (**E**,**F**) donors where recipients developed AKI. Significant differences in median FMN levels were found at all time points in (**A**).

FMN levels at individual time points during perfusion were similar between donors from whom a liver was utilized or not. Among all donors, peak FMN was significantly greater for the TA-NRP group (0.607 µg/mL [0.354–0.971] vs. 0.496 µg/mL [0.281–0.562], *p* = 0.042; Table 3). The increase in FMN during the first hour of perfusion was equally observed between A-NRP and TA-NRP (69.5% vs. 68.8%, *p* = 0.262). FMN did not correlate with lactate levels during perfusion. Among transplanted livers, FMN at all time points, as well as peak FMN, was significantly higher in donors with livers that developed EAD (e.g., 1.020 µg/mL [0.971–2.190] vs. 0.496 µg/mL [0.344–0.607], *p* < 0.001; Table 4 and Figure 2). Similarly, FMN at all time points, as well as peak FMN, was significantly positively correlated with the MEAF score (T0: rho = 0.327, *p* = 0.040; T1: rho = 0.514, *p* = 0.001; T2: rho = 0.432, *p* = 0.014; Peak: rho = 0.507, *p* = 0.001). Descriptively, donors with livers that developed biliary complications showed elevated levels of FMN throughout perfusion, as well as peak FMN (e.g., 0.933 µg/mL [0.728–1.210] vs. 0.500 µg/mL [0.344–0.702]; Table 4 and Figure 2). Among liver recipients who developed AKI, donor FMN levels did not differ from recipients without AKI (Table 4 and Figure 2). Delta FMN between T0 and T1, and T0 and T2, was not associated with post-transplant outcomes.

FMN was positively correlated with ALT during the first postoperative week and to a lesser extent to postoperative AST (Supplementary Table S2). FMN levels during perfusion did not correlate with post-transplant bilirubin and creatinine levels. Delta FMN between T0 and T1, and T0 and T2, did not correlate with postoperative laboratory values.

### 3.3. Lactate Levels During Perfusion and Association with Outcomes

Lactate at T0 was collected at 5 minutes [4–7] of perfusion, at T30 at 33 minutes [24–37], at T60 at 63 minutes [56–66], and at T90 at 89 minutes [80–94]. Mean lactate levels decreased during perfusion from 9.1 mmol/L (95% CI 8.5–9.7) at T0 to 6.7 mmol/L (95% CI 5.7–7.6) at T90 (Table 3). In donors with utilized livers, lactate levels decreased during the first hour of perfusion in 34 (85%), increased in 4 (10%), and were missing in 2 (5%). The increase in lactate was solely observed during A-NRP (24% vs. 0%, *p* = 0.032).

Descriptively, there was a trend toward lower mean lactate levels in donors with transplanted livers vs. non-utilized livers (6.2 mmol/L (95% CI 5.2–7.2) vs. 8.5 mmol/L (95% CI 5.4–11.5)) (Table 3 and Figure 1). Lactate was not associated with EAD or the MEAF score. Descriptively, T60 lactate levels were higher in donors with livers that developed biliary complications (9.0 mmol/L (95% CI 4.8–13.3) vs. 6.5 mmol/L (95% CI 5.6–7.3)) (Table 4 and Figure 2). Lactate levels during perfusion were not correlated with postoperative laboratory values (Supplementary Table S3). Delta lactate did not correlate with postoperative laboratory values.

### 3.4. Effect of Ischemic Periods on FMN and Lactate 

**Figure 3:**
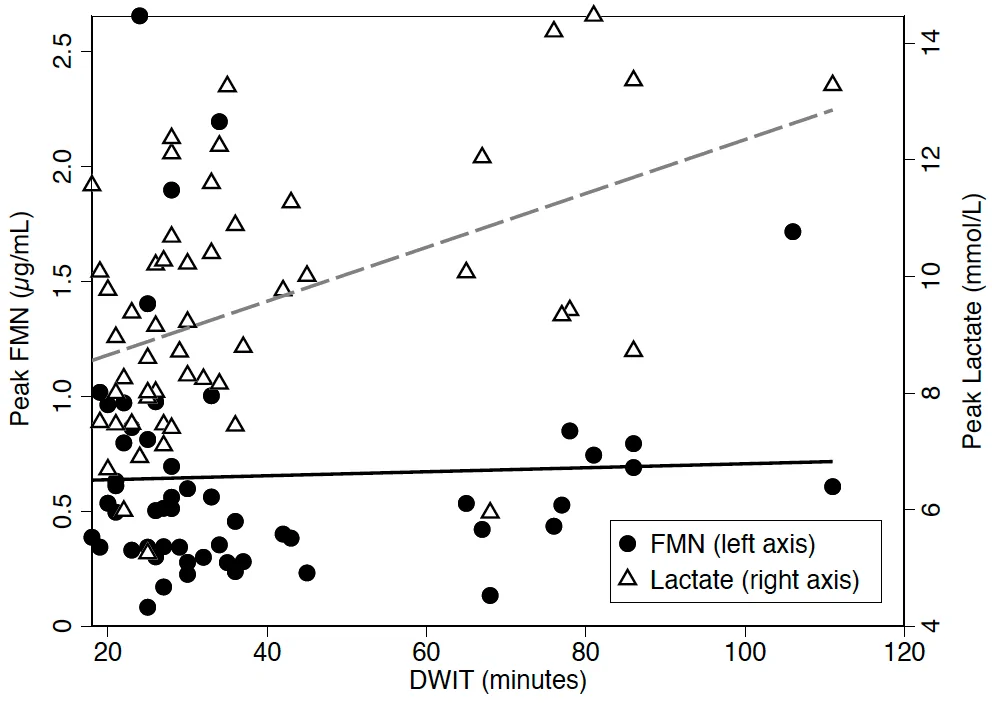
Correlation of peak FMN and peak lactate with donor warm ischemia time. Peak lactate increases significantly with longer donor warm ischemia time.

FMN levels at individual time points, as well as peak FMN and delta FMN during perfusion, did not differ between fDWIT < 20 min and ≥20 min, or DWIT < 30 min and ≥30 min (Table 5). However, lactate at individual time points, as well as peak lactate levels during perfusion, were significantly greater in donors with WIT ≥ 30 min compared to WIT < 30 min (Table 5 and Figure 3). Delta lactate was similar between the two groups. Compared to donors with a fDWIT < 20 min, mean, peak and delta lactate levels in donors with a fDWIT ≥ 20 min were similar.

## 4. Discussion

In this single-center exploratory study, perfusate levels of FMN and lactate during NRP were measured in 54 donors to assess changes during perfusion and their relationship with allograft outcomes. FMN levels increased during NRP and were generally greater among TA-NRP than A-NRP donors, likely reflecting a greater volume of tissue perfusion and the propensity of different tissues to release varying levels of FMN after an ischemic period. Lactate levels at all time points in our donor cohort were greater among donors who experienced a DWIT exceeding 30 min. Peak lactate levels were associated with duration of DWIT, supporting the concept that lactate may function as a marker to measure the immediate degree of metabolic derangement incurred by hypoxic injury during the agonal phase. FMN, unlike lactate, did not correlate with DWIT. In 40 transplanted livers, FMN detected immediately after the start of NRP and at subsequent time points during perfusion was associated with post-transplant transaminase levels, early allograft dysfunction and MEAF score. Lactate measured at any time point had no association with recipient graft outcomes. Because a consensus method to assess liver viability during NRP does not exist, our findings add to the nascent literature on the use of lactate and FMN as a measure of viability. Although not possible to discern in this small cohort with highly selected livers, one wonders if integrating FMN with lactate during liver graft assessment, particularly with longer duration of DWIT, might provide a synergistic measure of liver viability.

DCD liver transplantation with NRP-procured livers is limited by an inability to accurately assess risk accrued to the liver by a period of hypoxic warm ischemia during the donor agonal phase. In situ graft viability assessment has been described using a variety of methods, including overall appearance of the graft, bile production and biochemistry, perfusate levels of hepatocellular enzymes, perfusion flow characteristics, and lactate clearance from the perfusate [6,7,8]. Each of these methods has some basis for support, but also limitations. Lactate clearance is widely assessed but is limited by spillover into the perfusate from nonperfused ischemic donor tissue and extrinsically transfused blood during NRP [9]. Release of FMN into perfusate as a surrogate of ischemia–reperfusion injury has recently garnered attention as a potential biomarker for graft injury during ex situ liver perfusion [10,12,14,18,19,26,27]. The rapid expansion of ex situ machine perfusion has brought considerable interest in a variety of methods to determine graft viability, particularly among marginal organs such as steatotic livers and DCD livers with prolonged ischemic times [28]. Recent attention has focused on FMN in ex situ hypothermic machine perfusion (HOPE) and normothermic machine perfusion (NMP) as a promising biologic marker of ischemic injury with considerable predictive ability [10,12,18,19,26,27]. During HOPE, perfusate FMN has been associated with graft loss, patient survival, EAD, and cholangiopathy [10,18,19]. Similarly, perfusate FMN measurement during NMP may be helpful in graft selection, resulting in fewer overall complications, biliary complications, and improved graft survival [27].

Our analysis highlights several potential implications to measuring FMN from NRP perfusates. The first is that initial levels of FMN were strongly associated with EAD. This raises the possibility that measurement of FMN early during NRP could provide a meaningful assessment of liver viability. The development of FMN as a fluorometric test may facilitate the use of FMN as a point of care test at the time of NRP. Dingfelder et al. [19] described the ability of FMN measured immediately after cold reperfusion to predict EAD during HOPE, contributing to the idea that assessing liver injury can be done at a single initial time point rather than waiting for a dynamic trend as seen with lactate. Similarly, real-time assessment of FMN during HOPE and NMP has been shown to successfully guide decision-making [10,27]. While real-time assessment of FMN in ex situ perfusion of liver grafts is being performed, we experienced relevant complexities inherent to NRP. First of all, the duration of NRP is significantly shorter than ex situ perfusion, with 1 h of perfusion regarded as adequate in our program, and time pressure to make a decision on whether to utilize or not utilize the graft is greater. This drastically limits the ability to measure FMN on-site by fluorometry. An additional logistical issue for FMN testing is the location of the procurement, as this is generally done outside of the home institution hospital. A possible solution is to utilize donor care centers as a way to centralize DCD procurement or to develop a point-of-care FMN test.

To our knowledge, only one other group has examined FMN in the context of NRP. This was a single-center pilot study measuring FMN during perfusion in a cohort of 23 donors undergoing A-NRP [12]. NRP was conducted for 2 h, with FMN levels peaking at 30 min and then declining slightly thereafter. In contrast to our results, the authors described greater FMN levels in non-utilized livers and found no correlation with post-transplant transaminase levels. It is possible that the longer duration of donor asystolic time in this series compared to ours may account for the difference in FMN levels among utilized and non-utilized livers. This finding may also be explained by multiple factors inherent to NRP. In contrast to isolated liver perfusion in ex situ perfusion, multiple organs and whole donor compartments are being perfused with NRP. Therefore, FMN does not only originate from the liver, but may also leak from other mitochondria-rich organs and tissues that have suffered ischemic damage. Another potential contributor is the difference in perfusion area between TA-NRP and A-NRP. Our results point to higher FMN levels in donors undergoing TA-NRP as more tissue, as well as the mitochondrial-rich heart, are being perfused. Assessment of FMN should perhaps be different between these two modalities with A-NRP resembling a more isolated environment. We recommend investigating these differences in future studies with larger sample sizes that can accurately assess the impact of perfusion area on post-transplant results. With regard to ex-situ machine perfusion, FMN released during HOPE was significantly correlated to transaminases during the first postoperative week, but not to postoperative creatinine or bilirubin, reflecting a similar trend observed in our cohort [18].

Interestingly, FMN was not associated with the duration of DWIT or fDWIT in our study. Lactate, however, was greater at all time points in the DWIT ≥ 30 min group. This result is noteworthy because it is in line with the concept that lactate reflects hypoxic tissue damage. While one study has reported greater FMN values in WIT > 60 min vs. WIT < 30 min [11], this association has generally not been observed, despite being thought of as a major factor in FMN release.

Aside from the small sample size of this pilot study, there are several limitations to our analysis. NRP is an emerging practice in the United States and decisions as to which organs to accept or reject for transplant are evolving. As a result, selection bias is inevitable as some of the organs that we turned down when our NRP program was initiated would likely be transplanted at the present time, particularly those with longer DWIT. Additionally, perfusion duration during NRP is highly variable and depends on whether other organs are being procured, surgeon preference, or OPO practice. The small number of adverse outcomes limits a more robust statistically powered analysis. While we included biliary complications as an outcome, our cohort did not experience ischemic cholangiopathy which is regarded as the main risk of DCD liver transplantation [29]. Despite our small cohort with a subsequent absence of IC, the rationale behind including anastomotic strictures and bile leakages under the umbrella of biliary complications is the fact that the biliary epithelium suffers greatly from ischemia–reperfusion injury [30]. As such, we decided to include these complications that although may be partially due to operative factors, also represent the integrity of the biliary tract. Because of the small number of events, results with respect to biliary complications were presented in a descriptive manner and no statistical analyses were applied. However, this study is strengthened by consistent cannulation and perfusion strategies and by sampling of T0 and T1 FMN at almost identical time points during perfusion across donors, thereby lowering the inter-donor variability. This limits the performance bias generated by variation in a complex surgical environment. We therefore advocate for standardized decision-making regarding liver utilization in future studies to reduce the variability introduced by different practices, especially in multi-centered studies. Finally, as discussed by Dingfelder et al. [19], we reported FMN levels in concentrations rather than arbitrary units, thus being the first NRP study presenting absolute FMN values and subsequently improving the comparability of this data for future studies.

## 5. Conclusions

In summary, the data from this exploratory study suggest that detecting FMN levels during NRP is feasible and may have the potential to be associated with initial allograft function. Lactate did not show a clear association with clinical outcomes. Whether these findings can be replicated in a larger cohort remains to be determined.

## Figures and Tables

**Table 1:** Donor Characteristics.

	Total Cohort	Transplanted	Declined	*p*-Value
	*n* = 54	*n* = 40	*n* = 14	
Age	46 (38–55)	46 (38–54)	53 (37–59)	0.38
Sex				0.63
Male	30 (56%)	23 (57%)	7 (50%)	
Female	24 (44%)	17 (42%)	7 (50%)	
BMI	29 (25–34)	28 (24–32)	32 (28–44)	0.066
Race				**0.026**
White	44 (81%)	33 (82%)	11 (79%)	
Black	6 (11%)	6 (15%)	0 (0%)	
Asian	2 (4%)	0 (0%)	2 (14%)	
Hispanic	1 (2%)	0 (0%)	1 (7%)	
Missing	1 (2%)	1 (2%)	0 (0%)	
Cause of death				0.68
Anoxia	38 (70%)	27 (68%)	11 (79%)	
TBI	7 (13%)	5 (12%)	2 (14%)	
CVA	6 (11%)	5 (12%)	1 (7%)	
Other	3 (6%)	3 (8%)	0 (0%)	
ICU-LoS (days)	7 (5–9)	7 (5–10)	7 (6–8)	0.37
Comorbidities				
Diabetes	12 (22%)	7 (18%)	5 (36%)	0.16
Hypertension	28 (52%)	19 (48%)	9 (64%)	0.28
Alcohol	12 (22%)	11 (28%)	1 (7%)	0.11
Smoking	19 (35%)	16 (40%)	3 (21%)	0.21
Pre-withdrawal values				
Creatinine (mg/dL)	0.72 (0.52–1)	0.72 (0.51–0.96)	0.74 (0.61–1.60)	0.42
Bilirubin (mg/dL)	0.5 (0.3–0.75)	0.5 (0.3–0.6)	0.7 (0.6–1.2)	**0.013**
ALT (IU/L)	35 (18–74)	31 (20–65)	65 (22–99)	0.14
AST (IU/L)	45 (25–86)	42 (26–80)	68 (32–107)	0.20

Data are presented as median (p25, p75) for continuous, and *n* (%) for categorical data. TBI, traumatic brain injury; CVA, cerebrovascular accident; ICU-Los, intensive care unit-length of stay.The bold text represents significant *p*-values.

**Table 2:** Perfusion characteristics and ischemic times.

	Total Cohort	Transplanted	Declined	*p*-Value
	*n* = 54	*n* = 40	*n* = 14	
Agonal phase (min.)	18 (12–28)	16 (12–27)	19 (16–57)	0.14
Asystole duration (min.)	11 (9–15)	11 (10–13)	11 (9–17)	0.98
DWIT (min.)	29 (25–42)	28 (24–35)	36 (27–65)	0.14
DWIT				0.43
<30 min	28 (52%)	22 (55%)	6 (43%)	
≥30 min	26 (48%)	18 (45%)	8 (57%)	
fDWIT (min.)	20 (17–26)	19 (16–24)	24 (20–31)	**0.009**
fDWIT group				**0.030**
<20 min	25 (46%)	22 (55%)	3 (21%)	
≥20 min	29 (54%)	18 (45%)	11 (79%)	
NRP duration	114 (92–137)	114 (92–133)	112 (97–139)	0.64
Blood units administered	1 (0–3)	2 (0–3)	0 (0–1)	0.29
Total WIT (min.)		81 (76–88)		
Cold ischemia time (min.)		281 (227–322		
Reasons for graft declination				
High or rising lactate			8 (57%)	
Diseased appearance			3 (21%)	
Other			3 (21%)	

Data are presented as median (p25, p75) for continuous, and *n* (%) for categorical data. Agonal phase was defined as time from extubation to asystole. DWIT, donor warm ischemia time; fDWIT, functional donor warm ischemia time. fDWIT was defined as the time between mean arterial pressure less than 60 mmHg and start of perfusion. The bold text represents significant *p*-values.

**Table 3:** FMN and Lactate Levels *.

Lactate (mmol/L)		Total Cohort *n* = 54	Transplanted *n* = 40	Non-Utilized *n* = 14	*p*-Value	TA-NRP *n* = 31	A-NRP *n* = 23	*p*-Value
	T0	9.1 (2.2)	9.2 (2.3)	8.8 (1.7)	0.62	9.0 (2.0)	9.2 (2.4)	0.75
	T30	8.1 (2.6)	7.9 (2.6)	8.5 (2.8)	0.44	7.9 (2.4)	8.2 (2.9)	0.73
	T60	7.1 (2.6)	6.8 (2.6)	8.3 (2.7)	0.10	7.3 (2.5)	6.8 (2.8)	0.51
	T90	6.7 (3.0)	6.2 (2.6)	8.5 (3.6)	0.052	6.6 (2.7)	6.8 (3.3)	0.79
	Peak	9.5 (2.2)	9.4 (2.2)	9.6 (2.2)	0.80	9.2 (2.0)	9.8 (2.4)	0.39
FMN(µg/mL)								
	T0	0.308 (0.183–0.575)	0.308 (0.170–0.494)	0.336 (0.205–0.744)	0.42	0.406 (0.195–0.744)	0.240 (0.171–0.396)	0.18
	T1	0.443 (0.260–0.678)	0.426 (0.257–0.678)	0.583 (0.279–0.713)	0.47	0.534 (0.281–0.810)	0.396 (0.239–0.512)	0.099
	T2	0.486 (0.345–0.685)	0.486 (0.349–0.609)	0.545 (0.300–0.806)	0.75	0.545 (0.353–0.760)	0.456 (0.300–0.547)	0.16
	Peak	0.520 (0.344–0.797)	0.513 (0.345–0.804)	0.582 (0.281–0.744)	1.00	0.607 (0.354–0.971)	0.496 (0.281–0.562)	**0.042**

Data are presented as median (p25,p75) for non-normal data and mean (sd) for normal data. * Lactate levels were determined in 94% (51/54), 96% (52/54), 89% (48/54), and 70% (38/54) of the perfusate samples at T0, T30, T60, and T90, respectively. FMN was determined at all T0 time points, 51/54 (96%) of T1, and 44/54 (81%) of T2. The bold text represents significant *p*-values.

**Table 4:** Postoperative Complications by FMN and Lactate Levels.

Lactate (mmol/L)		Early Allograft Dysfunction *n* = 5	Immediate Graft Function *n* = 35	*p*-Value	Biliary Complications *n* = 4	*p*-Value *	No Biliary Complications *n* = 36	Acute Kidney Injury *n* = 13	No Acute Kidney Injury *n* = 27	*p*-Value
	T0	8.8 (2.5)	9.3 (2.3)	0.67	10.0 (2.3)		9.1 (2.3)	8.8 (2.0)	9.4 (2.4)	0.39
	T30	8.4 (1.2)	7.9 (2.8)	0.67	9.8 (2.7)		7.8 (2.6)	7.5 (2.6)	8.2 (2.7)	0.46
	T60	6.9 (1.5)	6.7 (2.7)	0.89	9.0 (2.7)		6.5 (2.5)	6.5 (2.8)	6.9 (2.5)	0.65
	T90	6.2 (1.2)	6.2 (2.8)	0.97	8.0 (2.8)		5.9 (2.5)	5.8 (3.0)	6.4 (2.4)	0.52
	Peak	9.4 (2.0)	9.4 (2.2)	0.96	10.2 (2.3)		9.3 (2.2)	8.9 (2.2)	9.7 (2.2)	0.28
FMN (µg/mL)										
	T0	0.849 (0.691–1.170)	0.266 (0.156–0.418)	**0.003**	0.592 (0.426–0.770)		0.270 (0.157–0.422)	0.274 (0.157–0.427)	0.318 (0.183–0.575)	0.58
	T1	1.020 (0.888–2.19)	0.396 (0.246–0.513)	**0.001**	0.731 (0.535–0.913)		0.411 (0.246–0.598)	0.450 (0.254–0.625)	0.420 (0.257–0.759)	0.39
	T2	0.760 (0.681–0.971)	0.451 (0.346–0.555)	**0.026**	0.721 (0.644–1.080)		0.443 (0.345–0.551)	0.547 (0.383–0.611)	0.451 (0.331–0.607)	0.39
	Peak	1.020 (0.971–2.190)	0.496 (0.344–0.607)	**0.001**	0.933 (0.728–1.210)		0.500 (0.344–0.702)	0.561 (0.383–0.611)	0.512 (0.344–0.971)	0.94

Data are presented as median (p25, p75) for non-normal data and mean (sd) for normal data. * Limited number of events and absence of ischemic cholangiopathy. The bold text represents significant *p*-values.

**Table 5:** FMN and Lactate Levels by ischemic times.

Lactate (mmol/L)		fDWIT < 20 min *n* = 25	fDWIT ≥ 20 min *n* = 29	*p*-Value	DWIT < 30 min *n* = 28	DWIT ≥ 30 min *n* = 26	*p*-Value
	T0	8.8 (2.3)	9.4 (2.0)	0.37	8.5 (1.9)	9.8 (2.3)	**0.029**
	T30	7.6 (2.8)	8.6 (2.5)	0.21	7.2 (2.2)	9.2 (2.7)	**0.002**
	T60	6.4 (2.6)	7.7 (2.5)	0.093	6.2 (2.1)	8.0 (2.9)	**0.016**
	T90	6.1 (2.9)	7.3 (3.0)	0.23	5.3 (2.2)	8.2 (3.0)	**0.001**
	Peak	9.1 (2.3)	9.8 (2.0)	0.22	8.6 (1.8)	10 (2.2)	**0.002**
FMN (µg/mL)							
	T0	0.344 (0.196–0.494)	0.265 (0.171–0.575)	0.88	0.331 (0.148–0.671)	0.270 (0.195–0.480)	0.97
	T1	0.411 (0.257–0.667)	0.484 (0.281–0.701)	0.42	0.512 (0.302–0.759)	0.423 (0.246–0.651)	0.36
	T2	0.496 (0.346–0.722)	0.476 (0.300–0.607)	0.58	0.503 (0.387–0.760)	0.421 (0.278–0.681)	0.21
	Peak	0.527 (0.344–0.812)	0.513 (0.302–0.695)	0.84	0.548 (0.345–0.914)	0.446 (0.281–0.690)	0.19

Data are presented as median (p25,p75) for non-normal data, and mean (sd) for normal data. DWIT, donor warm ischemia time; fDWIT, Functional donor warm ischemia time. fDWIT was defined as the time between mean arterial pressure less than 60 mmHg and start of perfusion. The bold text represents significant *p*-values.

## Data Availability

The datasets presented in this article are not publicly available. The data are part of an ongoing study. Requests to access the datasets should be directed to the corresponding author.

## Supplementary Materials

The following supporting information can be downloaded at: https://www.mdpi.com/article/10.3390/medicina62091622/s1, Table S1. Recipient Characteristics; Table S2. Spearman Correlations between FMN and Postoperative Laboratory Levels; Table S3. Spearman Correlations between Lactate and Postoperative Laboratory Levels.

## Abbreviations

The following abbreviations are used in this manuscript:

A-NRP	Abdominal normothermic regional perfusion
AKI	Acute kidney injury
ALT	Alanine aminotransferase
AS	Anastomotic strictures
AST	Aspartate aminotransferase
BL	Biliary leakage
DCD	Donation after circulatory death
DWIT	Donor warm ischemia time
EAD	Early allograft dysfunction
FMN	Flavin mononucleotide
fDWIT	Functional donor warm ischemia time
HOPE	Hypothermic machine perfusion
IQR	Interquartile range
IRB	Institutional review board
MEAF	Model for early allograft function
NMP	Normothermic machine perfusion
NRP	Normothermic regional perfusion
OPO	Organ procurement organization
TA-NRP	Thoracoabdominal normothermic regional perfusion
