# Supplementary material for: The Association of Flavin Mononucleotide and Lactate During Normothermic Regional Perfusion with Post-Transplant Liver Outcomes: An Exploratory Study
Source: Medicina (Kaunas). 2026 Aug 22;62(9):1622. doi: 10.3390/medicina62091622 (PMC13609822; doi:10.3390/medicina62091622)
Supplement: Supplementary file 1 [file medicina-62-01622-s001.zip › Supplementary_Table S1.pdf]

Supplementary Table S1. Recipient Characteristics

**N=40**	
**Age (years)**	58 (50-64)
**Sex**	
Male	16 (40%)
Female	24 (60%)
**BMI (kg/m²)**	28 (26-33)
**MELD-Na**	23 (16-26)
**Race**	
White	28 (70%)
Black	2 (5%)
Asian	4 (10%)
Other	4 (10%)
Missing	2 (5%)
**Primary Diagnosis**	
Alcohol	17 (42%)
MASH	8 (20%)
HCC	4 (10%)
Viral Hepatitis	2 (5%)
Autoimmune	1 (2%)
Other	8 (20%)
**Complications**	
Early allograft dysfunction	5 (13%)
Model for early allograft function	3.16 (1.52)
Biliary complications	4 (10%)
Anastomotic strictures	3 (8%)
Bile leakage	1 (3%)
Acute kidney injury	13 (33%)
Median follow-up time (days)	456 (209 – 644)

Data are presented as median (p25,p75) for continuous, and n (%) for categorical data.
