# Supplementary material for: The Association of Flavin Mononucleotide and Lactate During Normothermic Regional Perfusion with Post-Transplant Liver Outcomes: An Exploratory Study
Source: Medicina (Kaunas). 2026 Aug 22;62(9):1622. doi: 10.3390/medicina62091622 (PMC13609822; doi:10.3390/medicina62091622)
Supplement: Supplementary file 1 [file medicina-62-01622-s001.zip › Supplementary_Table S2.pdf]

Supplementary Table S2. Spearman Correlations between FMN and Postoperative Laboratory Levels

	FMN T0	FMN T1	FMN T2		FMN T0	FMN T1	FMN T2
**ALT POD1**	0.34*	0.51**	0.45*	**Bilirubin POD1**	0.19	0.17	0.27
**ALT POD2**	0.33*	0.52***	0.44*	**Bilirubin POD2**	0.07	0.16	0.22
**ALT POD3**	0.37*	0.53***	0.39*	**Bilirubin POD3**	0.08	0.19	0.28
**ALT POD4**	0.36*	0.50**	0.39*	**Bilirubin POD4**	0.12	0.17	0.29
**ALT POD5**	0.35*	0.45**	0.34	**Bilirubin POD5**	0.11	0.18	0.28
**ALT POD6**	0.27	0.40*	0.33	**Bilirubin POD6**	0.08	0.13	0.23
**ALT POD7**	0.30	0.40*	0.37*	**Bilirubin POD7**	0.08	0.12	0.2
**AST POD1**	0.49*	0.50**	0.40*	**Creatinine POD1**	-0.22	-0.19	-0.18
**AST POD2**	0.30	0.42**	0.32	**Creatinine POD2**	-0.32*	-0.29	-0.23
**AST POD3**	0.30	0.35*	0.30	**Creatinine POD3**	-0.09	-0.09	-0.08
**AST POD4**	0.28	0.30	0.38	**Creatinine POD4**	-0.13	-0.07	-0.05
**AST POD5**	0.33*	0.21	0.23	**Creatinine POD5**	-0.13	-0.02	0.02
**AST POD6**	0.13	0.11	0.27	**Creatinine POD6**	-0.03	0.03	0.07
**AST POD7**	0.09	0.01	0.17	**Creatinine POD7**	0.16	0.13	0.19

*0.01<p<0.05, **0.001<p<0.01, ***p<0.001
POD, postoperative day; ALT, alanine aminotransferase; AST, aspartate aminotransferase
