# Supplementary material for: The Association of Flavin Mononucleotide and Lactate During Normothermic Regional Perfusion with Post-Transplant Liver Outcomes: An Exploratory Study
Source: Medicina (Kaunas). 2026 Aug 22;62(9):1622. doi: 10.3390/medicina62091622 (PMC13609822; doi:10.3390/medicina62091622)
Supplement: Supplementary file 1 [file medicina-62-01622-s001.zip › Supplementary_Table S3.pdf]

Supplementary Table S3. Spearman Correlations between Lactate and Postoperative Laboratory Levels									
	**Lactate T0**	**Lactate T30**	**Lactate T60**	**Lactate T90**		**Lactate T0**	**Lactate T30**	**Lactate T60**	**Lactate T90**
**ALT POD1**	0.10	0.22	0.29	0.34	**Bilirubin POD1**	-0.02	0.09	-0.04	-0.05
**ALT POD2**	0.08	0.22	0.22	0.29	**Bilirubin POD2**	0.04	0.02	-0.08	-0.12
**ALT POD3**	0.09	0.24	0.14	0.24	**Bilirubin POD3**	0.09	0.01	-0.08	-0.13
**ALT POD4**	0.13	0.29	0.14	0.21	**Bilirubin POD4**	0.05	-0.04	-0.16	-0.21
**ALT POD5**	0.14	0.29	0.11	0.20	**Bilirubin POD5**	-0.03	-0.08	-0.16	-0.13
**ALT POD6**	0.09	0.23	0.11	0.23	**Bilirubin POD6**	0.01	-0.07	-0.17	-0.14
**ALT POD7**	0.18	0.33 *	0.19	0.27	**Bilirubin POD7**	0.05	-0.08	-0.21	-0.16
**AST POD1**	0.07	0.24	0.31	0.35	**Creatinine POD1**	-0.13	-0.24	-0.22	-0.32
**AST POD2**	0.13	0.24	0.24	0.26	**Creatinine POD2**	-0.11	-0.25	-0.20	-0.15
**AST POD3**	0.15	0.30	0.16	0.29	**Creatinine POD3**	-0.09	-0.17	-0.20	-0.15
**AST POD4**	0.20	0.32 *	0.11	0.11	**Creatinine POD4**	-0.02	-0.13	-0.16	-0.14
**AST POD5**	0.14	0.22	0.01	0.12	**Creatinine POD5**	0.02	-0.07	-0.10	-0.18
**AST POD6**	0.03	0.08	0.01	0.17	**Creatinine POD6**	0.04	-0.05	-0.05	-0.21
**AST POD7**	0.17	0.24	0.11	0.20	**Creatinine POD7**	0.04	0.01	0.05	-0.22

*0.01<p<0.05, **0.001<p<0.01, ***p<0.001. POD, postoperative day; ALT, alanine aminotransferase; AST, aspartate aminotransferase
